# Prostate cancer detection with transrectal in-bore MRI biopsies: impact of prostate volume and lesion features

**DOI:** 10.1186/s13244-025-01942-6

**Published:** 2025-03-23

**Authors:** Alexander Schaudinn, Harald Busse, Constantin Ehrengut, Nicolas Linder, Jonna Ludwig, Toni Franz, Lars-Christian Horn, Jens-Uwe Stolzenburg, Timm Denecke

**Affiliations:** 1 Department of Diagnostic and Interventional Radiology, University Hospital Leipzig, Leipzig, Germany; 2 Department of Diagnostic and Interventional Radiology, Section of Pediatric Radiology, University Medical Center Hamburg-Eppendorf, Hamburg, Germany; 3 Division of Radiology and Nuclear Medicine, HOCH Health Ostschweiz, St. Gallen, Switzerland; 4 Department of Urology, University Hospital Leipzig, Leipzig, Germany; 5 Institute of Pathology, University of Leipzig, Leipzig, Germany; 6 Present Address: Center of Radiology and Nuclear Medicine (ZRN) Leipzig, Leipzig, Germany

**Keywords:** Prostate cancer, Biopsy, Magnetic resonance imaging, In-bore, Image guidance

## Abstract

**Objectives:**

To systematically analyze the diagnostic outcome of transrectal in-bore MRI-guided biopsies as a function of prostate volume and lesion features.

**Methods:**

This single-center study retrospectively included 184 consecutive patients with transrectal in-bore MRI biopsies and histological analysis after multiparametric MRI diagnostics of at least one PI-RADS ≥ 3 lesion. Diagnostic and biopsy MRI data were analyzed for a number of patient and imaging features, specifically prostate volume, lesion size, lesion location (longitudinal, sagittal and segmental) and lesion depth. Features were then compared for statistically significant differences in the cancer detection rate (CDR) of clinically significant (cs-PCa) and any prostate cancer (any-PCa) using categorical and continuous variables.

**Results:**

A total of 201 lesions were biopsied detecting cs-PCa in 26% and any-PCa in 68%, respectively. In subgroup analyses of all features, the CDR of cs-PCa differed significantly between ranges of lesion size only (*p* < 0.001, largest for large lesions). In multivariable analysis, however, only PI-RADS score and PSA showed a significant association with a higher risk of cs-PCa.

**Conclusions:**

The cancer detection rates of transrectal in-bore MRI-guided biopsies did not vary significantly for prostate volume, lesion size or lesion location. This suggests that the diagnostic performance of such an approach is not necessarily compromised for challenging biopsy settings like large glands, small lesions or eccentric locations. A translation of these findings to other cohorts might be limited by the low detection rate for clinically significant cancer.

**Critical relevance statement:**

This systematic analysis indicates that the diagnostic performance of transrectal in-bore biopsies might not be substantially impaired by patient-specific factors like prostate volume, lesion size, and lesion location, making it a viable option for challenging biopsy cases as well.

**Key Points:**

- The impact of prostate and lesion features on in-bore MRI biopsy performance is controversial.
- Neither prostate volume, lesion size, nor location showed significant impact on cancer detection.
- In-bore biopsy does not seem to be limited by challenging sampling geometries.

**Graphical Abstract:**

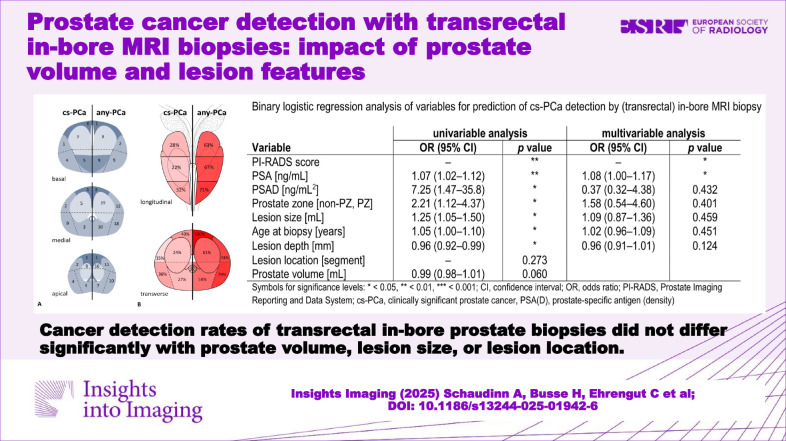

## Introduction

The diagnostic workup of prostate cancer (PCa) has evolved substantially over the last decade. Currently, there is level 1A evidence that the addition of multiparametric MRI (mpMRI) and subsequent MRI-guided biopsy increases the detection of clinically significant prostate cancer (cs-PCa) when compared to systemic ultrasound-guided biopsy alone [1, 2]. An MRI pathway reduces the number of clinically insignificant (indolent) cancers as well as the number of biopsies needed. Prebiopsy mpMRI is therefore widely endorsed in international guidelines [3]. The choice of performing biopsy (PI-RADS scores ≥ 3) under MRI guidance alone or in combination with systematic biopsies has been widely discussed. Recent data compared against whole-mount histopathology [4], however, suggested that the combination was superior to both individual techniques alone with a higher detection rate for cs-PCa and less upgrades of the cancer grade group.

Despite the additional effort, the demand for MRI-guided biopsies is likely to grow. At the technical level, three different approaches can be distinguished: in-bore MRI-guided biopsy (short: in-bore biopsy), MRI/ultrasound fusion-guided biopsy (short: fusion biopsy), and cognitive biopsy. In prospective analyses, no method has so far proven to be superior when it comes to the detection of cs-PCa [5, 6]. Recent retrospective studies, however, suggest that in-bore biopsies might be more accurate than fusion biopsies [7, 8]. In-bore biopsies do not require prior image registration and have the advantage that intraprocedural MRI controls the advance of the biopsy needle to the target area and documents the exact sampling position [9].

Little is known about the impact of features like prostate volume, lesion size, and lesion location on the performance of these biopsy techniques and the literature is scarce. One prospective study has found no impact but was limited by sample size and a categorical analysis of subgroups [6]. In contrast, Venderink et al have observed significantly higher detection rates for in-bore biopsies over fusion biopsies, particularly in smaller lesions (up to 8 mm) [10].

Most of the available data comes from in-bore biopsies, yet again, results are highly variable. For smaller lesions, some authors have reported lower cancer detection rates [11, 12] while others did not [10, 13]. Schouten et al have observed that significant cancers tend to be missed in the apical and dorsolateral prostate segments (when compared with transrectal ultrasound (TRUS) biopsy) [14] and others have found that detection rates will be significantly lower in larger prostates [11, 15]. Between these studies, methods were clearly different with regard to patient selection (PI-RADS 3-5 vs. 4-5), number of target lesions (index only vs. multiple ones), measurement procedure (lesion size), or statistical assessment (categorical versus continuous variables).

It is therefore difficult to suggest an optimal biopsy technique for an (highly) individual geometry of the lesion and gland—considering, for instance, the differences between a small eccentric lesion in a large gland and a large midline midgland tumor close to the rectal wall. Further insight into the strengths and weaknesses of each biopsy technique—in our case an in-bore approach—is needed to support the interdisciplinary decision (on a biopsy technique) and to ultimately improve procedural performance and outcome. The aim of this study was therefore to systematically analyze the diagnostic outcome of transrectal in-bore biopsies as a function of common gland and lesion features.

## Materials and methods

### Patients

This single-center retrospective study originally included 224 consecutive patients (264 lesions) who had all undergone in-bore MRI biopsy between June 2010 and August 2022 at our institution. Patient referral was based on an elevated prostate-specific antigen level (PSA > 4 ng/mL) or abnormal digital rectal examination (DRE), either with or without previous prostate biopsy, and during follow-up on active surveillance. Indication for biopsy was an interdisciplinary decision using all imaging and clinical information, such as PSA serum level, PSA density and their progression. Patients after focal treatment of a known prostate cancer were excluded from this retrospective data analysis (six patients with seven lesions). Patients with at least one prostate lesion with a PI-RADS score ≥ 3 (according to corresponding PI-RADS versions or v2.1 for ten cases that predated v1) were identified [16–18] and consistently reevaluated by the same expert reader with v2.1 only. This led to the further exclusion of 34 patients with 56 lesions after downgrade (PI-RADS < 3), leaving 184 final patients with 201 lesions. The study was approved by the institutional review board, and written informed consent was obtained from all patients.

### Multiparametric MRI

Prebiopsy mpMRI was mainly performed in-house (174/184 patients) at 3 T (Magnetom Trio or Prisma Fit, Siemens Healthcare, Erlangen, Germany, and Ingenia 3.0 T, Philips Healthcare, Best, The Netherlands) using a combination of phased-array pelvic and spine coils. In 47 patients (between June 2010 and June 2015), an additional endorectal coil had been used. The MRI protocol underwent minor adjustments over the study period but always complied with the respective PI-RADS criteria at the time of scanning. PI-RADS technical standards were also met for ten external referrals and ten MRI examinations that predated PI-RADS v1. Our current diagnostic protocol is summarized in supplementary Table S1.

### Image analysis

**Fig. 1:**
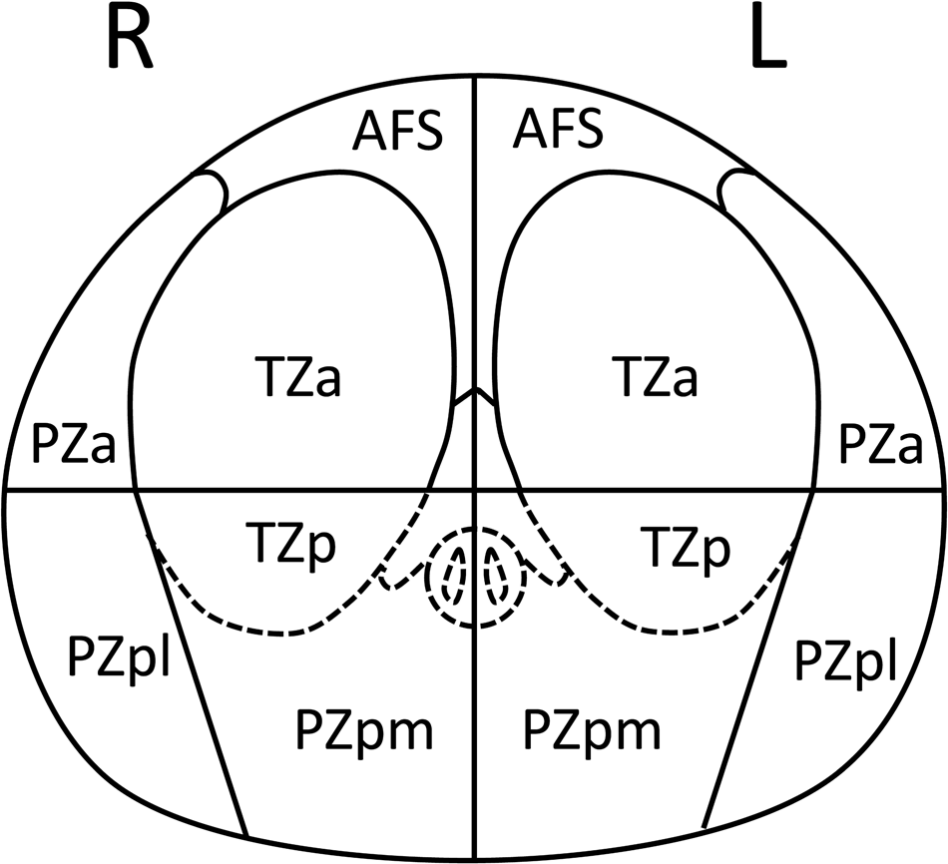
Anatomical segments derived from the PI-RADS v2.1 sector map of the prostate (transverse view) for the right (R) and left (L) gland side: AFS, anterior fibromuscular stroma; PZa, anterior peripheral zone; PZpl, posterior lateral peripheral zone; PZpm, posterior medial peripheral zone; TZa, anterior transition zone; TZp, posterior transition zone. Notes: PZpm and TZp form one anatomical segment. For further statistical analysis, corresponding right and left side segments were summed

**Fig. 2:**
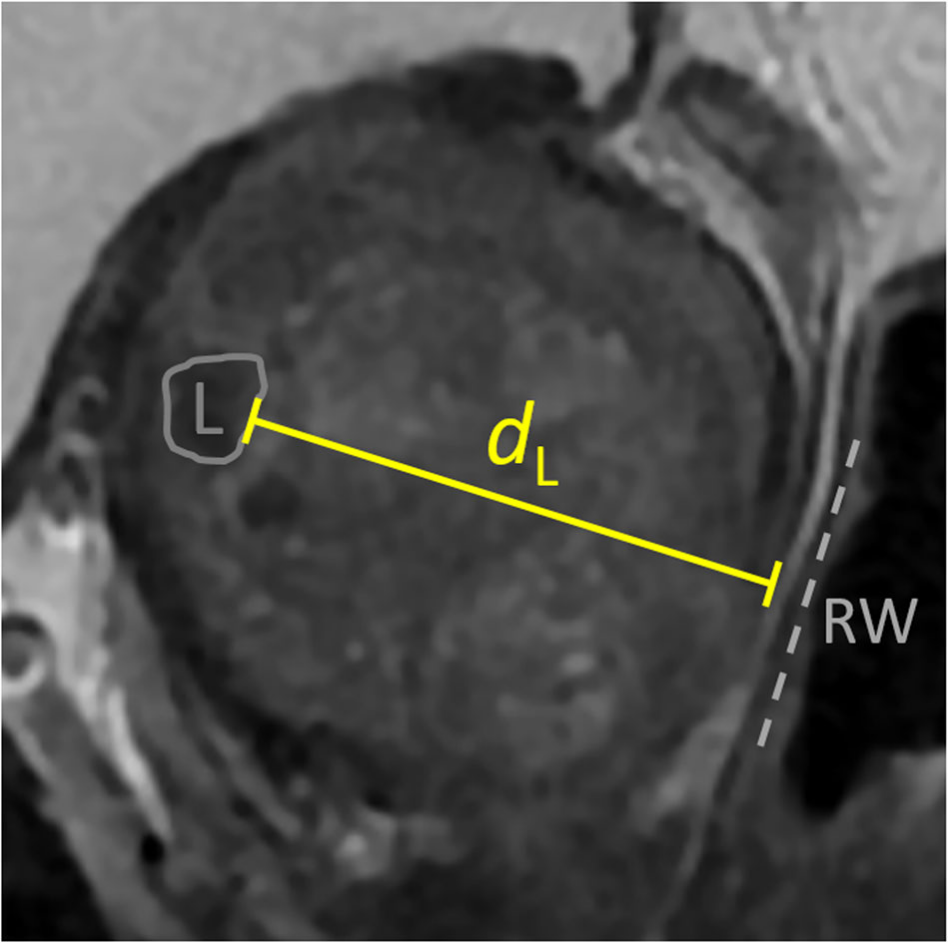
Illustration of parameter lesion depth *d*_L_, which is measured in a sagittal T2w image as the (minimal) distance between the lesion (L) and the posterior prostate surface—perpendicular to a line adjusted to the rectal wall (RW). In this example, the prostate is enlarged (92 mL), the lesion is small (0.7 mL) and located in the anterior transition zone at midgland level. This translates to a longer needle path, also indicated by a larger *d*_L_ (38 mm). Lesions in contact with the posterior prostate surface have a *d*_L_ of 0 mm

Up to three index lesions were analyzed per patient. Images were originally read in consensus by radiologists with at least three years of experience with urogenital MRI and/or mpMRI using the historical PI-RADS version. For the present analysis, MRI data were retrospectively read according to version 2.1 (v2.1) only by one board-certified uroradiologist (A.S.) with then 10 years of experience with prostate mpMRI. During that review, PI-RADS scores were adjusted for 101 lesions (5 upgrades and 96 downgrades, 70 patients), resulting in the exclusion (v2.1 score of 2) of 56 lesions (34 patients). Lesions were annotated in T2w images and assigned to one of the ten prostate segments defined in PI-RADS v2.1 [12] (Fig. 1). PI-RADS also standardized the way to measure lesion size as the largest diameter for routine assessment [12]. In this study, lesion and prostate diameters were annotated in all three dimensions (axial and sagittal images), and respective volumes were computed with the ellipsoid formula (0.5236 × height × width × depth). A novel quantity, lesion depth, was defined in a sagittal T2w image as the (minimal) distance between lesion and posterior prostate surface (perpendicular to the rectal wall, Fig. 2). Throughout the study, MRI readers were unaware of the histopathological biopsy results.

### MRI-guided biopsy

MRI-guided in-bore biopsies were performed in an outpatient setting at 3 T or 1.5 T (Magnetom 3 T Trio or 1.5 T Aera, Siemens Healthcare, Erlangen, Germany). All patients received prebiopsy antibiotics with the latest protocol using a single intravenous shot of Ertapenem (1 g, Invanz, Merck Sharp & Dohme B.V., Harleem, The Netherlands) 30 min prior to biopsy. With the patient in prone position, a needle guide was inserted into the rectum and connected to an interventional device (151 cases with DynaTRIM, Invivo, Gainesville, FL, USA, and 33 cases with RCM, Soteria Medical, Arnhem, The Netherlands). Intraprocedural challenges like the limited range for needle guide adjustments or prostate indentation were addressed by positioning the patient (pads/cushions) according to his size and needle deviation from the midplane, and by careful insertion/retraction of the needle guide, respectively [19]. Biopsy planning involved axial and sagittal T2w TSE (turbo spin-echo) or T2w/T1w TRUFI images (true fast imaging with steady-state free precession).

Prostate features were correlated between diagnostic (typically ADC and T2w) and intraprocedural images to visually reidentify the target lesion. The needle guide was then adjusted toward the target of interest (manually/via remote control). If the lesion could not be reliably identified by T2w imaging, axial diffusion-weighted imaging data were acquired as well. After proper alignment of the needle guide, the patient was moved out of the bore for manual needle insertion. Biopsy cores were taken with an MRI-compatible, fully automatic biopsy gun (18G, needle length 150 or 175 mm, Invivo), which allowed us to reach distant anterior lesions as well. Control images with the needle in place were taken for all cases and both targeting approaches (manual/remote control). This involved axial and sagittal T2w images, typically along the needle axis, to document the sampling location. Sampling continued until the number and position of the taken samples were (subjectively) deemed adequate by an experienced radiologist independent of the respective lesion size or location. Systematic biopsies were not performed.

### Pathological analysis

All biopsy cores underwent evaluation by a senior pathologist (19 years of experience in urogenital pathology) according to the standards of the Consensus Conference of the International Society of Urological Pathology (ISUP; 2005, 2014 and 2019, respectively) [20–22]. The pathologist had standard clinical information like PSA level or DRE status but was blinded to the MRI findings. Biopsy cores were reported for ISUP grade/Gleason score. Clinically significant prostate cancer was defined as at least one core containing ISUP grade 2 (Gleason 3 + 4) cancer or higher.

### Statistical analysis

Descriptive statistics were used to present population characteristics and in-bore biopsy results. CDR analysis with categorical variables involved subgroups of prostate volume (< 30, 30–60, 60–90, ≥ 90 mL), lesion size (< 0.25, 0.25–0.5, 0.5–1.0, ≥ 1.0 mL) and lesion depth (< 10, 10–20, 20–30, ≥ 30 mm). Number and boundaries were chosen according to previous reports [10, 11] or common values. Given the gland’s bilateral symmetry, individual numbers for lesions identified on the corresponding left and right segments were added to obtain larger numbers for statistical testing (see also Fig. 1). In addition, cancer detection rates were analyzed by multivariable binary logistic regression, including those variables that were significant in univariable analysis. Continuous and categorical variables were reported as median, while frequencies were reported as percentages (rounded to full numbers).

Significance was generally assumed for *p* < 0.05; degrees (*p* ranges) were denoted by asterisk symbols (* < 0.05, ** < 0.01, *** < 0.001). Pearson’s chi-square test was used to test the independence of proportions (CDR) between independent samples (categories), mainly between different ranges of a given geometric parameter (see above). Confidence intervals (95%) for CDR were estimated by bootstrap (1000 samples, simple). All statistical analyses were performed with SPSS (Version 27, IBM Corp., Armonk, NY).

## Results

### Patient and lesion characteristics

A total of 201 MRI-targeted lesions were biopsied in 184 men. The majority of them, 124 (67%), had previously undergone at least one negative prostate biopsy, 57 (31%) were biopsy naïve and three (2%) were on active surveillance for a low-risk PCa (ISUP grade 1). Main baseline patient and lesion characteristics, including zonal lesion location, are shown in Table 1.

**Table 1:** Patient and lesion characteristics

Number of patients	184
Age (years)	67 (53–85)
Number (percentage) of biopsy-naïve patients	57 (31%)
Number (percentage) of patients with prior negative biopsy	124 (67%)
TRUS biopsy	113 (91%)
In-bore biopsy	9 (7%)
Fusion biopsy	2 (2%)
Number (percentage) of patients on active surveillance	3 (2%)
PSA (ng/mL)	8.5 (0.7–42.0)
Prostate volume (mL)	48.3 (13.4–270.9)
PSA density (ng/mL^2^)	0.18 (0.03–1.36)
Number of MRI-targeted lesions	201
PI-RADS 3	51 (25%)
PI-RADS 4	66 (33%)
PI-RADS 5	84 (42%)
Lesion size (mL)	0.76 (0.05–13.1)
Sagittal lesion depth (mm)	11 (0–52)
Location, zonal	
Peripheral zone	117 (58%)
Transition zone	78 (39%)
Anterior fibromuscular stroma	5 (2%)
Central zone	1 (< 1%)

All values are given as a median (range) unless stated otherwise

*Fusion biopsy* MRI/ultrasound fusion-guided biopsy, *in-bore biopsy* in-bore MRI-guided biopsy, *PCa* prostate cancer, *PSA* prostate-specific antigen, *PI-RADS* Prostate Imaging Reporting and Data System, *TRUS biopsy* transrectal ultrasound-guided biopsy

### Biopsy results

In total, 629 biopsy cores were analyzed by histopathology (median 3 cores per target lesion), revealing any-PCa in 59% (374/629) of the cores. At the lesion level, cs-PCa was found in 26% (53/201) and any-PCa in 68% (136/201). At the patient level, 27% of men (50/184) were diagnosed with cs-PCa and 70% (128/184) with any-PCa. The median time of the biopsy procedure was 39 min (range: 12 to 124 min), including 13 cases with two and two cases with three separate target lesions, respectively. Detailed biopsy results are shown in Table 2.

**Table 2:** Biopsy results

Number of biopsy cores taken	629
Number of biopsy cores taken per lesion	3 (1–9)
Number (percentage) of PCa-positive biopsy cores	374 (59%)
Number of PCa-positive biopsy cores per lesion	2 (0–7)
Histopathological findings per lesion	
Prostate cancer, ISUP Grade Group	136 (68%)
1	83 (41%)
2	32 (16%)
3	15 (7%)
4	5 (3%)
5	1 (< 1%)
Atypical small acinar proliferation (ASAP)	2 (1%)
High-grade prostatic intraepithelial neoplasia (hgPIN)	1 (< 1%)
Benign finding	62 (31%)

All values are given as a median (range) unless stated otherwise

*PCa* prostate cancer, *ISUP* International Society of Urological Pathology

### Prostate cancer detection

Overall detection rates for cs-PCa and any-PCa divided into specific (prebiopsy) PI-RADS scores are given in Table 3. In univariable logistic regression, PI-RADS score, PSA, PSAD, prostate zone, lesion size, age and lesion depth showed a significant association with the presence of cs-PCa. Only PI-RADS score and PSA remained significant in the multivariable regression model with these seven variables (see Table 4). The number of cores taken per lesion was not correlated with the detection rate of cs-PCa in univariable and multivariable analysis.

**Table 3:** Cancer detection rates (in %, with confidence intervals CI in brackets) according to PI-RADS categories (patient numbers in parentheses)

PI-RADS	cs-PCa	any-PCa
3	0% (0/51)	12% [4–22%] (6/51)
4	21% [11–32%] (14/66)	74% [63–85%] (49/66)
5	46% [36–57%] (39/84)	96% [92–100%] (81/84)
3 + 4 + 5	26% [20–33%] (53/201)	68% [61–74%] (136/201)
4 + 5	35% [28–43%] (53/150)	87% [81%–92%] (130/150)

*PI-RADS* Prostate Imaging Reporting and Data System, *PCa* prostate cancer, *cs-PCa* clinically significant prostate cancer

**Table 4:** Binary logistic regression analysis of variables for prediction of cs-PCa detection by (transrectal) in-bore MRI biopsy

	Univariable analysis		Multivariable analysis	
Variable	OR (95% CI)	*p*-value	OR (95% CI)	*p*-value
PI-RADS score	–	**	–	*
PSA (ng/mL)	1.07 (1.02–1.12)	**	1.08 (1.00–1.17)	*
PSAD (ng/mL^2^)	7.25 (1.47–35.8)	*	0.37 (0.32–4.38)	0.432
Prostate zone (non-PZ, PZ)	2.21 (1.12–4.37)	*	1.58 (0.54–4.60)	0.401
Lesion size (mL)	1.25 (1.05–1.50)	*	1.09 (0.87–1.36)	0.459
Age at biopsy (years)	1.05 (1.00–1.10)	*	1.02 (0.96–1.09)	0.451
Lesion depth (mm)	0.96 (0.92–0.99)	*^.^	0.96 (0.91–1.01)	0.124
Lesion location (segment)	–	0.273		
Prostate volume (mL)	0.99 (0.98–1.01)	0.060^.^		

Symbols for significance levels: * < 0.05, ** < 0.01

*CI* confidence interval, *OR* odds ratio, *PI-RADS* Prostate Imaging Reporting and Data System, *cs-PCa* clinically significant prostate cancer, *PSA(D)* prostate-specific antigen (density)

The CDR values between subgroups of gland and lesion features and PI-RADS scores 3-5 in categorical assessment are shown in Table 5. Lesion size was the only variable that showed a statistically significant difference (*p* < 0.01) for the CDR of cs-PCa in subgroup analyses.

**Table 5:** Cancer detection rates (in %, with confidence intervals CI in brackets) of suspicious prostate lesions (PI-RADS 3-5) as a function of prostate volume and lesion features (patient numbers in parentheses)

Features	cs-PCa	any-PCa
Prostate volume (mL)		
< 30	22% [7–39%] (6/27)	81% [67–95%] (22/27)
30; 60	31% [23–40%] (34/109)	76% [68–83%] (83/109)
60; 90	26% [13–41%] (10/39)	51% [35–67%] (20/39)
≥ 90	12% [0–26%] (3/26)	42% [23–65%] (11/26)
*p*-value	0.212	***
Lesion size (mL)		
< 0.25	17% [6–29%] (6/36)	53% [36–70%] (19/36)
0.25; 0.5	15% [4–29%] (5/33)	45% [29–64%] (15/33)
0.50; 1.0	17% [6–28%] (8/48)	73% [60–85%] (35/48)
≥ 1.0	40% [30–51%] (34/84)	80% [71–87%] (67/84)
*p*-value	**	***
Sagittal lesion depth (mm)		
< 10	33% [24–43%] (30/90)	74% [65–83%] (67/90)
10; 20	23% [14–33%] (17/74)	70% [60–81%] (52/74)
20; 30	19% [5–37%] (5/26)	58% [39–76%] (15/26)
≥ 30	9% [0–30%] (1/11)	18% [0–50%] (2/11)
*p*-value	0.167	**
Location, axial		
Basal	27% [15–41%] (13/48)	60% [47–75%] (29/48)
Medial	22% [14–31%] (20/90)	69% [59–79%] (62/90)
Apical	32% [21–44%] (20/63)	71% [61–86%] (45/63)
*p*-value	0.417	0.444
Location, sagittal		
Anterior	22% [15–31%] (24/109)	67% [58–75%] (73/109)
Posterior	32% [22–41%] (29/92)	68% [59–78%] (63/92)
*p*-value	0.128	0.820
Location, segment		
PZpl	36% [22–51%] (17/47)	79% [66–90%] (37/47)
PZpm+TZp	27% [14–39%] (12/45)	58% [44–72%] (26/45)
PZa	15% [5–29%] (5/34)	74% [59–88%] (25/34)
TZa	24% [15–34%] (17/70)	61% [50–71%] (43/70)
AFS	40% [0–100%] (2/5)	100% [100–100%] (5/5)
*p*-value	0.254	0.066

Symbols for significance levels: ** < 0.01, *** < 0.001 (Pearson chi-square)

*PI-RADS* Prostate Imaging Reporting and Data System, *PCa* prostate cancer, *cs-PCa* clinically significant prostate cancer, *AFS* anterior fibromuscular stroma, *PZa* anterior peripheral zone, *PZpl* posterior lateral peripheral zone, *PZpm* posterior medial peripheral zone, *TZa* anterior transition zone, *TZp* posterior transition zone

**Fig. 3:**
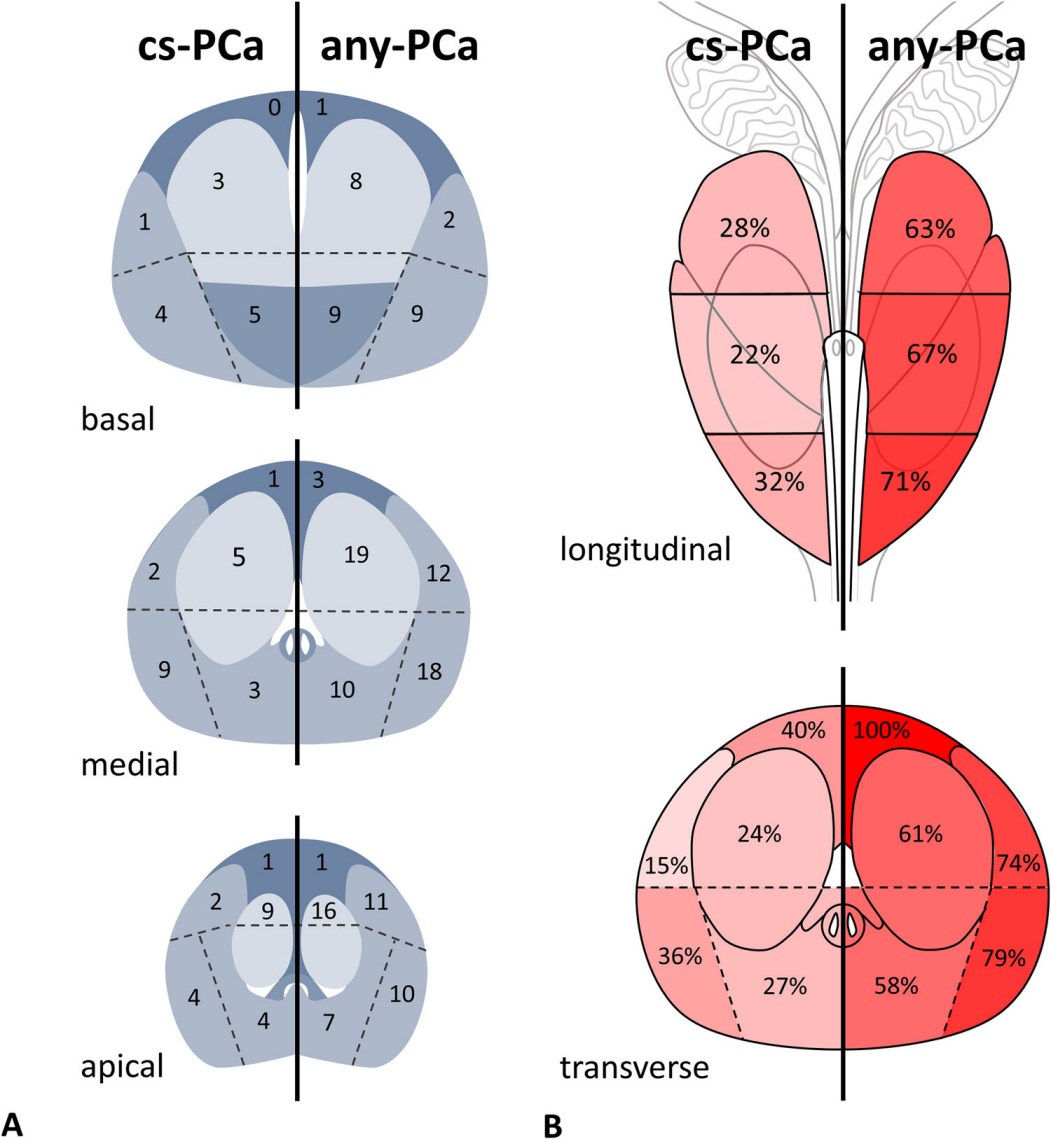
Distribution of cancer-positive segments according to PI-RADS sector map after transrectal in-bore MRI biopsy. **A** Transverse distributions of actual number of findings (left: cs-PCa, right: any-PCa) for the basal, medial and apical level of the prostate, respectively. **B** Longitudinal distribution of cancer detection rate (percent) for all levels after adding numbers over all transverse segments (top) and transverse distribution of cancer detection rate (percent) for all segments after adding numbers over all longitudinal levels (bottom)

Figure 3A shows the numbers of detected cancers per segment. Regarding the main location of the lesion, CDR differences for cs-PCa and any-PCa were neither significant in craniocaudal direction (base vs. midgland vs. apex, Fig. 3B) nor between anterior and posterior parts of the gland. Figure 3B also shows the distribution of CDR for any-PCa and cs-PCa for individual segments (no distinction between left and right, nor craniocaudal location) with nonsignificant differences again.

## Discussion

This work has retrospectively analyzed a relatively large number of in-bore transrectal prostate biopsies from a single institution. The main goal was to compare the potential impact of different gland and lesion features of PI-RADS 3-5 findings on diagnostic outcome (cancer detection rate, CDR). In a multivariable logistic regression analysis of prostate volume, lesion size, segmental location and lesion depth, none of these image features, but PI-RADS score and PSA value, were significant. In categorical subgroup analysis of these image features, only lesion size showed a significant difference in the CDR of clinically significant prostate cancer (cs-PCa), with the highest CDR observed for larger volumes.

So far, only a few studies have looked specifically at the diagnostic outcome of in-bore biopsies as a function of lesion size. In multivariable analysis, most studies, including this one, have not observed a statistically significant impact of lesion size on the CDR of cs-PCa [12, 13, 15, 23, 24]. In contrast, simple categorical subgroup analyses have predominantly reported an effect [11, 12, 25] rather than not [10] but often lacked statistical testing. In line with our findings, Schimmöller et al, have seen a significant difference in the CDR of cs-PCa between lesion size subgroups (highest CDR for largest lesions) but did not perform a multivariable analysis [11]. In summary, we regard lesion size as less of a factor in the diagnostic performance of in-bore biopsies and have not seen a poorer performance for very small lesions. It should also be stressed that—although a common descriptive parameter—lesion size is still reported in different ways across the corresponding literature: as simply the largest diameter (following the PI-RADS standard [16]), as two (or three) diameters on axial (and additional sagittal or coronal) MR images or, as in our study, as an actual volume (ellipsoidal approximation).

The median prostate volume of our cohort (48 mL) was similar to volumes reported in the literature [7, 13, 24–27], with a substantial proportion (about 13%) of volumes even larger than 90 mL. Categorical as well as multivariable analysis did not show a significant impact of prostate volume on the CDR of cs-PCa. This finding is in line with current studies using multivariate analyses [12, 23, 28]. In-bore biopsies might therefore be an option for larger gland volumes, considering that ultrasound-guided techniques (transperineal or transrectal) have shown poorer results in distant, particularly anterior locations and larger glands [14, 29–31].

Two previous analyses of in-bore biopsies have found a significantly lower CDR of cs-PCa for larger prostate volumes [11, 15]. In one case, this might be explained by a categorical analysis only and different inclusion criteria (PI-RADS 4-5 versus 3-5) [11], whereas disagreement in the second case remains unclear [15]. This highlights the need for further investigation, ideally as a direct comparison between biopsy techniques, in particular the most common one (fusion) and the most elaborate one (in-bore).

Regarding the segmental location of the biopsy targets (PI-RADS sector map), Schouten et al have concluded that significant cancers in the apical and dorsolateral segments tend to be missed by in-bore biopsy [14]. In contrast, our CDR analysis for cs-PCa did not reveal any significant difference between segments—neither in categorical nor in multivariable logistic regression analysis. The CDR in our dorsolateral segments (PZpl) was actually higher than the median CDR of all segments (36% vs. 27%). Besides that, Schouten et al did not provide a *p*-value nor lesion size, which might be a confounder [14].

There is a general lack of distinct segmental CDR analyses of cs-PCa based on the PI-RADS sector map. Some authors have considered six zones for the entire gland [13], while others simply distinguished between three (base, midgland and apex) [28, 32] or two (peripheral and transition zone) [12, 24], with neither of them reporting significant differences. Similarly, we found no proof of a higher CDR in the anterior parts of the gland, as indicated by several antecedent studies, that often lacked statistical testing [6, 14, 23, 28]. Finally, our newly introduced lesion depth showed no statistical effect in multivariable logistic regression. In summary, our results provide further evidence that the diagnostic performance of in-bore biopsies does not depend on lesion location.

Our study has a number of limitations. The detection rates for cs-PCa were remarkably low (26%). Although similar CDRs have been reported for several other analyses as well (25–35%) [6, 11, 15, 28, 33–35], this limits the degree to which our findings (no impact of studied features) may hold implications for other cohorts. In contrast to some of the highly controlled trials [1], our analysis was retrospective, single-center and gathered data over a longer period of time. This brings along procedural variability (imaging, biopsy, and pathology) and lower statistical validity.

At the diagnostic level, MRI technology has evolved. All foci were reevaluated by just one uroradiologist who used the latest PI-RADS version for study inclusion to minimize reader bias. At the biopsy level, we have not excluded very small or multiple lesions, unlike other studies [10, 24, 28, 36], which should have effectively increased their CDR per lesion. In-bore biopsies, in general, are prone to sampling errors from partial volume averaging or susceptibility artifacts. Pathology was assessed by mainly one experienced uropathologist but lacks reference workup for ISUP grading and correlation with radical prostatectomy results. Similar to many MRI-targeted approaches we have no information on false-negative results, which could simply arise from lesions invisible on diagnostic MRI. Drost et al have reported the fraction of men with mpMRI-negative cs-PCa to be about 9% [37]. At the statistics level, some authors have restricted their computation to lesions with a PI-RADS score of 4-5 (excluding 3) [7, 10, 11], which should give a higher CDR.

In parts we used data from MR imaging assisted by an endorectal coil, which for its distortional effect on the prostate potentially influenced our gland and lesion measurements. The resulting changes in prostate volumes, however, may be considered low [38]. Finally, procedural complications have not been assessed systematically across the study period.

In conclusion, our logistic regression analysis of transrectal in-bore biopsies did not reveal any significant differences in the detection rate of clinically significant cancer when considering multiple variables like prostate volume, lesion size or segmental location. This suggests that the diagnostic performance of a transrectal in-bore approach is not necessarily compromised by challenging biopsy settings like large glands, small lesions, or eccentric locations. A translation of these findings to other cohorts might be limited by the low detection rate for clinically significant cancers.

## Supplementary information

ELECTRONIC SUPPLEMENTARY MATERIAL

## Data Availability

The datasets used and/or analyzed during the current study are available from the corresponding author upon reasonable request.

## Abbreviations

any-PCa: Any degree prostate cancer
CDR: Cancer detection rate
CI: Confidence interval
cs-PCa: Clinically significant prostate cancer
ISUP: International Society of Urological Pathology (Consensus Standard)
mpMRI: Multiparametric MRI
OR: Odds ratio
PCa: Prostate cancer
PI-RADS: Prostate imaging reporting and data system
T1w/T2w: T1/T2-weighted imaging
TRUS: Transrectal ultrasound
