# Supplementary material for: Prostate cancer detection with transrectal in-bore MRI biopsies: impact of prostate volume and lesion features
Source: Insights Imaging. 2025 Mar 23;16:69. doi: 10.1186/s13244-025-01942-6 (PMC11930903; doi:10.1186/s13244-025-01942-6)
Supplement: Supplementary file 1 — ELECTRONIC SUPPLEMENTARY MATERIAL [file 13244_2025_1942_MOESM1_ESM.pdf]

Prostate cancer detection with transrectal in-bore MRI biopsies: impact of prostate volume and lesion features
ELECTRONIC SUPPLEMENTARY MATERIAL

Supplementary Table s1

Imaging parameters of multiparametric prostate MRI

	Sequence	Slices	ST [mm]	IPR [mm]	TR [ms]	TE [ms]	FOV [mm]	FA [°]
T2w (tra, cor, sag)	TSE	25-26	3.0.	0.4-0.7	4490	101-108	220 x 220	120
DWI (tra)	EPI	20	3.0	1.6 x 1.6	3000	58	220 x 220	90
T1w DCE (tra)	GRASP	24	3.0	1.1 x 1.1	4.0	1.86	240 x 240	12

DWI-diffusion weighted imaging; DCE-dynamic contrast enhanced; TSE-turbo spin echo; SS-EPI-single-shot echo planar imaging; SPGR-spoiled gradient echo; ST-slice thickness; IPR-in-plane resolution; TR-repetition time; TE-echo time; FOV-field of view; FA-flip angle.
